# Subtype-Specific Surface Proteins on Adipose Tissue Macrophages and Their Association to Obesity-Induced Insulin Resistance

**DOI:** 10.3389/fendo.2022.856530

**Published:** 2022-04-11

**Authors:** Kristina Strand, Natalie Stiglund, Martha Eimstad Haugstøyl, Zahra Kamyab, Victoria Langhelle, Laurence Lawrence-Archer, Christian Busch, Martin Cornillet, Iren Drange Hjellestad, Hans Jørgen Nielsen, Pål Rasmus Njølstad, Gunnar Mellgren, Niklas K. Björkström, Johan Fernø

**Affiliations:** ^1^ Hormone Laboratory, Department of Medical Biochemistry and Pharmacology, Haukeland University Hospital, Bergen, Norway; ^2^ Mohn Nutrition Research Laboratory, Department of Clinical Science, University of Bergen, Bergen, Norway; ^3^ Center for Infectious Medicine, Department of Medicine Huddinge, Karolinska Institutet, Karolinska University Hospital, Stockholm, Sweden; ^4^ Plastikkirurg1, Bergen, Norway; ^5^ Department of Medicine, Haukeland University Hospital, Bergen, Norway; ^6^ Department of Surgery, Voss Hospital, Haukeland University Hospital, Bergen, Norway; ^7^ Center for Diabetes Research, Department of Clinical Science, University of Bergen, Bergen, Norway; ^8^ Department of Pediatrics and Adolescents, Haukeland University Hospital, Bergen, Norway

**Keywords:** surface proteomics, adipose tissue macrophage, obesity, insulin resistance, flow cytometry

## Abstract

A chronic low-grade inflammation, originating in the adipose tissue, is considered a driver of obesity-associated insulin resistance. Macrophage composition in white adipose tissue is believed to contribute to the pathogenesis of metabolic diseases, but a detailed characterization of pro- and anti-inflammatory adipose tissue macrophages (ATMs) in human obesity and how they are distributed in visceral- and subcutaneous adipose depots is lacking. In this study, we performed a surface proteome screening of pro- and anti-inflammatory ATMs in both subcutaneous- (SAT) and visceral adipose tissue (VAT) and evaluated their relationship with systemic insulin resistance. From the proteomics screen we found novel surface proteins specific to M1-like- and M2-like macrophages, and we identified depot-specific immunophenotypes in SAT and VAT. Furthermore, we found that insulin resistance, assessed by HOMA-IR, was positively associated with a relative increase in pro-inflammatory M1-like macrophages in both SAT and VAT.

## Introduction

Obesity is a major global health problem associated with high risk of developing co-morbidities, such as insulin resistance and type 2 diabetes (T2D) (1). Obesity-induced inflammation, which is typically chronic and low-grade, is considered to be a mechanistic link between obesity and insulin resistance (2, 3). The inflammation is manifested by accumulation of immune cells in the adipose tissue (AT), and elevated levels of pro-inflammatory cytokines such as tumor necrosis factor (TNF), C-C motif chemokine ligand (CCL) 2, and interleukin (IL)-6 that can block insulin signaling through various mechanisms (4).

Obesity-related adipose tissue inflammation is in large driven by infiltration of monocytes from the circulation that differentiates into pro-inflammatory macrophages (5, 6). Pro-inflammatory macrophages may also be derived from anti-inflammatory macrophages already present in the tissue that undergo a phenotypic switch in response to the stressed adipose tissue microenvironment (7, 8). Pro-inflammatory M1 macrophages are normally involved in clearing pathogens during infections while the anti-inflammatory M2 macrophages have functions in tissue repair. These subsets of macrophages can be identified by different surface receptors, such as CD80, CD86, CD206, and CD163 (9, 10). However, the pro- and anti-inflammatory macrophages located in the adipose tissue express somewhat different surface receptors, and they are therefore normally referred to as “M1-like” and “M2-like” macrophages (11, 12). In humans, the M1-like macrophages are typically characterized by co-expression of CD11c and CD206, and tend to gather around necrotic adipocytes, forming syncytial aggregates known as crown-like structures (CLS) (11). Indeed, the CLS have been demonstrated to be associated with insulin resistance in human obesity (13). The M2-like macrophages, on the other hand, express CD206 but not CD11c, and are normally dispersed throughout the tissue (11).

Individuals with central obesity that accumulate fat in the visceral adipose tissue (VAT) rather than in subcutaneous adipose tissue (SAT) generally have an increased risk for obesity-related metabolic disease (14–16). These individuals typically display higher levels of inflammation and infiltration of macrophages in their VAT (17, 18). However, other studies report that insulin resistance is associated with elevated amounts of macrophages in SAT instead of VAT (11, 13). Thus, the exact role of pro- and anti-inflammatory macrophages in SAT compared to VAT with respect to metabolic disease remains controversial. Furthermore, in recent years the M1-like/M2-like paradigm has been challenged by the identification of other pro-inflammatory adipose tissue macrophages (ATMs), the so-called metabolically activated macrophages (Mme’s), identified in adipose tissue of both rodents and humans (19, 20). These macrophages are activated by nutritional signals including glucose, insulin, and fatty acids and express surface receptors such as CD36, ABCA1, and PLIN2 that differ from the ones expressed by M1- and M2-like macrophages (19). Thus, the immunological landscape of adipose tissue and the difference between various adipose tissue depots is still not fully understood, and there is currently no established consensus regarding which surface receptors best define pro- and anti-inflammatory M1- and M2-like ATMs or the novel MMe’s in humans.

To better characterize the adipose tissue M1- and M2-like macrophages in humans and investigate their role in obesity-induced insulin resistance, we here analyzed M1- and M2-like macrophages in SAT and VAT in a sizeable cohort of individuals with obesity. We then performed a surface proteome characterization of M1- and M2-like macrophages and AT monocytes from human SAT in comparison to blood monocytes and validated key findings in another cohort of individuals with obesity. Next, we investigated how the levels of M1- and M2-like macrophages related to the degree of insulin resistance. Lastly, we explore how the macrophages related to other inflammatory features, such as the formation of CLS and AT pro-inflammatory gene expression. The results are discussed in relation to current knowledge about the links between inflammation and obesity-induced insulin resistance.

## Material and Methods

### Clinical Cohorts and Study Subject Details

The patient cohorts used in this study was approved by the Regional Committees for medical and health research ethics (REK 2015/2343 and REK 2010/502) and written informed consent was obtained from all participants. This study included two clinical cohorts consisting of individuals with obesity undergoing bariatric surgery at Voss Regional Hospital and with patients undergoing plastic surgery. The first cohort consisted of 57 individuals and was used for flow cytometry characterization of ATMs, gene expression and immunohistochemistry analysis. The second cohort contained 23 individuals and was used for verification of surface protein expression by flow cytometry. Clinical characteristics and biochemical measurements of the patients are presented in **Tables 1** and **2**. HOMA-IR was calculated with the HOMA2 calculator using fasting glucose and insulin levels (21). Liposuction aspirates from individuals undergoing plastic surgery were used for the surface proteome screening of ATMs. Buffy coats from anonymous donors were used both as controls and as blood samples for the surface proteome screening. The buffy coats were obtained from the Blood bank services as Haukeland University Hospital.

**Table 1:** Individuals with obesity – cohort 1.

Subjects	57
T2D	10/57
Gender	70.2% females (40/57)
Age	44.0 (22 – 70)
BMI (kg/m^2^)	41.5 (31.1 – 57.4)
Glucose (mmol/L)	5.70 (4.70 – 8.10)
Insulin (mIU/L)	15.1 (3.20 – 50.5)
C-peptide (nmol/L)	1.31 (0.480 – 2.74)
HbA1c (mmol/mol)	37.0 (26.0 – 54.0)
HOMA-IR	1.98 (0.415 – 6.62)
Total cholesterol (mmol/L)	4.70 (2.50 – 6.40)
HDL-C (mmol/L)	1.10 (0.500 – 2.80)
LDL-C (mmol/L)	3.10 (1.10 – 4.30)
Triglycerides (mmol/L)	1.32 (0.530 – 5.94)
CRP (mg/L)	4.00 (0.240 – 25.0)

Data are given as median (range), T2D, type 2 diabetes; BMI, body mass index; HOMA-IR, homeostatic model assessment of insulin resistance index; LDL-C, low-density lipoprotein cholesterol; HDL-C, high-density lipoprotein cholesterol; CRP, C-reactive protein

**Table 2:** Individuals with obesity– cohort 2.

Subjects	23
Gender	78.2% females (18/23)
Age	35.0 (22 – 64)
BMI (kg/m^2^)	42.1 (29.3 – 54.0)
Glucose (mmol/L)	5.50 (4.40 – 10.9)
Insulin (mIU/L)	13.5 (3.50 – 36.2)
C-peptide (nmol/L)	0.95 (0.040 – 1.58)
HbA1c (mmol/mol)	34.0 (27.0 – 62.0)
HOMA-IR	1.74 (0.531 – 4.57)
Total cholesterol (mmol/L)	4.20 (3.00 – 7.40)
HDL-C (mmol/L)	1.20 (0.800 – 1.60)
LDL-C (mmol/L)	2.70 (1.50 – 5.30)
Triglycerides (mmol/L)	1.19 (0.650 – 2.03)
CRP (mg/L)	6.00 (1.00 – 34.0)

Data are given as median (range), T2D, type 2 diabetes; BMI, body mass index; HOMA-IR, homeostatic model assessment of insulin resistance index; LDL-C, low-density lipoprotein cholesterol; HDL-C, high-density lipoprotein cholesterol; CRP, C-reactive protein.

### Isolation of PBMC From Blood Samples

PBMC were isolated from heparin blood samples or buffy coats using density gradient centrifugation. The blood sample was diluted in PBS (Sigma Aldrich), carefully layered on top of Lymphoprep (Stemcell Technologies) and centrifuged at 2000 rpm for 20 minutes with brake and acceleration set to 1. The leukocyte layer was isolated and washed in PBS and the cells were counted before staining for flow cytometry.

### Isolation of Stromal Vascular Cells From Adipose Tissue Biopsies or Liposuction Aspirates

Stromal vascular cells (SVCs) were isolated from adipose tissue biopsies or liposuction aspirates. Adipose tissue biopsies from the subcutaneous and the visceral adipose tissue were collected during bariatric surgery and kept in Krebs Ringer Phosphate (KRP) buffer until further processing. Biopsies were cut into smaller pieces before enzymatic digestion with collagenase type I (Life Technologies, 0.66 mg/ml). Liposuction aspirates were washed in NaCl to get rid of excess blood and diluted in the same volume of Krebs Ringer Phosphate (KRP) buffer as the volume of the fat, before enzymatic digestion by Liberase (Roche, final concentration 0,078 Wünsch units/ml). Digestion was carried out for about 1 h with shaking at 37°C. The digested tissue samples were then filtered, and the stromal vascular cells were removed from underneath the floating layer of mature adipocytes. The SVCs were washed in PBS and centrifuged at 300 x *g* for 5 minutes to pellet the stromal vascular cells. The SVCs isolated from the liposuction aspirates were treated with red blood cell lysis buffer. The cells were counted, and freshly isolated SVCs were stained immediately for flow cytometry analysis.

### Flow Cytometry

Macrophage surface protein characterization and verification of surface protein expression was performed on freshly isolated PBMC and SVC samples. Antibodies used are listed in **Table S1** LIVE/DEAD Fixable Aqua Dead Cell Stain kit (Invitrogen, 1:100 dilution) was used to distinguish between live and dead cells during analysis. Staining was performed in FACS buffer (PBS with 2 mM EDTA and 2% FBS) for 20 min at RT in the dark. Following staining, the cells were washed twice in FACS buffer before the cells were fixed in 2% formaldehyde for 15 minutes. After washing twice, the cells were resuspended in FACS buffer and kept at 4°C in the dark.

Surface proteome screening was performed using the LEGENDScreen™ Human PE Kit (Biolegend, Cat# 700007). First, barcoding was performed to be able to separate PBMCs and SVC in the analysis. PBMCs were thawed and stained with CD45 BV650 (Cat# 304044), while the freshly isolated SVC were stained with CD45 AF700 (Cat# 304024). The PBMC and SVC were mixed and stained with a backbone panel containing the following antibodies: BB515 CD206 (Cat# 564668) from BD Biosciences, APC-Cy7 HLA-DR (Cat# 307617) and BV605 CD14 (Cat# 301834) from Biolegend and PE-Cy5.5 CD11c (Cat# MHCD11c18) from Life Technologies. The backbone staining was performed for 20 min at room temperature (RT) in the dark before washing. The cells were then added to the plates provided in the LEGENDScreen™ kit and the rest of the experiment was performed according to the manufacturer’s protocol.

All samples were run on an 18-color LSR Fortessa (BD Biosciences) with 407, 488, 561 and 640 lasers using the BD FACSDiva™ Software (BD Biosciences). Flow cytometry data was analyzed using FlowJo version 10 (Treestar, USA) with the DownSample and Uniform Manifold Approximation and Projection (UMAP) plugins. Acquired data was compensated in FlowJo using a compensation matrix generated based on antibody-stained control beads. For UMAP analysis, the different myeloid populations from blood, SAT or VAT were concatenated, generating 8 files. These files were then downsampled so that all samples contained the same number of events. The files were then concatenated for analysis. UMAP was run using all parameters from the verification panel.

### Gene Expression Analysis

Total RNA was isolated from frozen adipose tissue using the RNA/DNA/Protein Purification Plus kit (Norgen Biotek Corp.). Briefly, approximately 100 mg of adipose tissue was cut, and the tissue was lysed in Buffer SKP (from the kit) using cold stainless-steel beads in a Tissuelyser (Qiagen) for 3 x 2 minutes at 25 Hz. RNA was then purified according to the protocol provided in the kit. cDNA was synthesized using 350 ng RNA input with the High-Capacity cDNA Reverse Transcription Kit (Applied Biosystems) and diluted 1:2 in PCR-grade water. Real-time qPCR was performed using SYBR Green I Master (Roche) on a Lightcycler ^®^ 480 II (Applied Biosystems). Primers were purchased from Sigma (see **Table S2**). Gene expression was calculated relative to the expression of importin 8 (IPO8).

### Immunohistochemistry and Determination of Adipocyte Cell Size

Adipose tissue biopsies collected during bariatric surgery were kept in Histocon transport solution (Histolab) before the tissue was fixed in 4% formalin (in PBS) for approximately 24 h. The tissue was then transferred to 70% EtOH before paraffin embedment, sectioning and staining with CD68. Antigen unmasking and deparaffinization were performed simultaneously using a PTLink machine (Dako, Denmark) containing Target Retrieval Solution, Low pH (Dako, Denmark). The sections were heated at 98°C for 24 minutes and cooled down to 58°C. The sections were then washed in PBS and incubated with 3% H_2_O_2_ (in H_2_O) for 10 minutes to block endogenous peroxidase activity, before washing again. The sections were then incubated with 2-3 drops of INNOVEX Background Buster (Biosciences, #NB306) for 20 minutes in a moisture chamber at RT. Following washing, the sections were stained with the primary antibody mouse-anti human CD68 (Thermo Fisher Scientific Cat# 14-0688-82) at a concentration of 0.05 mg/ml diluted in Normal Horse Serum 2.5% (ImmPRESS kit, Vector, MP-7402) over night at 4°C. Mouse anti-human IgG1 antibody (Abcam, ab18443) was used as isotype control. Following primary antibody staining, the sections were washed and incubated with 2-3 drops of the secondary antibody, horse anti-mouse IgG conjugated with HRP (ImmPress kit, Vector MP-7402) for 30 minutes at room temperature. After washing, the sections were incubated with DAB substrate (Vector, SK-4105) diluted in ImmPACT DAB diluent (Vector, SK-4105) for 10 minutes at RT before washing again. The sections were then counterstained with hematoxylin for 3 minutes before they were rehydrated in increasing concentrations of EtOH (70, 96 and 100%) and xylene. Lastly, the sections were mounted with cover glass using Pertex glue (Histolab. The slides were imaged using an Olympus BX61VS microscope with the imaging system Olympus VS120 S6 slide scanner. CD68+ macrophages in crown-like structures around adipocytes were identified. Crown-like structures were defined three or more macrophages surrounding an adipocyte (11). The images were processed in Image J (Fiji) with the plugin Adiposoft to measure adipocyte diameter (22). The median diameter was calculated for each sample.

### Statistical Analysis

Data was analyzed using Prism version 9 (GraphPad). Correlation analyses were performed using SPSS (IBM) or R (https://www.r-project.org). D’Agostino & Pearson omnibus normality test was used to determine normality of the data. For normally distributed data, the t-test or one-way ANOVA with Tukey’s multiple comparisons test was used. When data was not normally distributed, the Mann-Whitney t-test or Kruskal-Wallis test with Dunn’s multiple comparisons test was used. Correlation analyses in SPSS were performed on log-transformed data using bootstrapped (bias-corrected and accelerated, BCa) confidence intervals. Details on statistical analysis are given in figure legends together with the number of subjects included in each analysis. A p-value of < 0.05 was considered statistically significant.

## Results

### Visceral Adipose Tissue Contains More M1-Like Macrophages Than Subcutaneous Adipose Tissue

**Figure 1:**
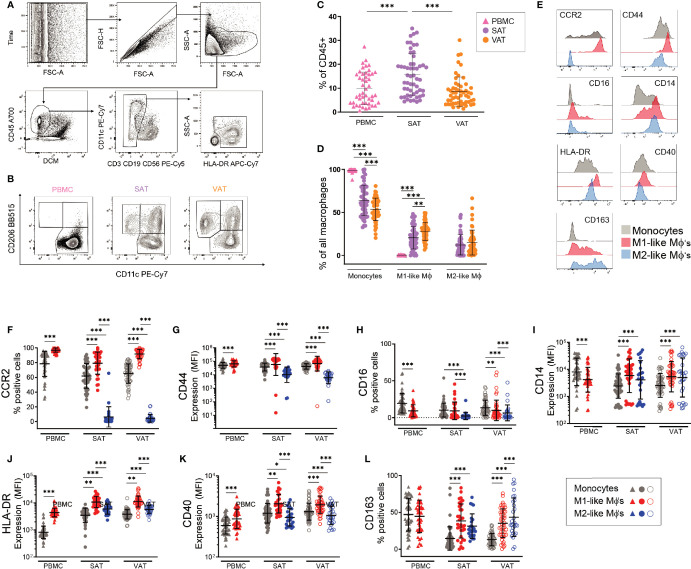
Identification and characterization of macrophage populations in blood and adipose tissue of humans with obesity. **(A)** Flow cytometry gating scheme used to identify monocytes and macrophages. Arrows indicate the sequence of gating. **(B)** Representative flow cytometry plots showing CD206 and CD11c expression on monocytes (CD11c+CD206-), M1-(CD11c+CD206+) and M2-like (CD206+CD11c-) macrophages from PBMC, SAT, and VAT samples from one patient. **(C)** The total monocyte and macrophage population as a fraction of total CD45+ cells in PBMC (n=47), SAT (n=54), and VAT (n=54). **(D)** Monocytes, M1- and M2-like macrophages (MΦ) as a fraction of the total monocyte and macrophage population in PBMC (n=47), SAT (n=54), and VAT (n=54). **(E)** Representative stainings for the surface proteins shown in **(F-L)**. **(F-L)** Scatter plots showing expression of surface proteins **(F)** CCR2, **(G)** CD44, **(H)** CD16, **(I)** CD14, **(J)** HLA-DR, **(K)** CD40, and **(L)** CD163 on monocytes (grey), M1 (red)-, and M2 (blue)-like macrophages (MΦ’s) from PBMC (n=47), SAT, and VAT (n=54). For **(F, H, L)** scatter plots showing percentage of cells expressing the proteins. For **(G, I-K)** scatter plots showing expression (mean fluorescence intensity, MFI) of surface proteins. For **(C, D, F-L)** line and error bars represent mean and SD. For **(C, D)**, the Mann-Whitney *U*-test was used for comparison between groups. **p< 0.01, ***p<0.001. For **(E-L)**, the Wilcoxon matched pair signed rank test was used for comparison between groups, *p < 0.05, **p < 0.001, ***p < 0.001.

We first set out to compare macrophage levels in SAT and VAT and to characterize adipose tissue M1- and M2-like macrophage composition in a depot-specific manner. Using flow cytometry, we analyzed stromal vascular cells from both SAT and VAT as well as peripheral blood mononuclear cells (PBMC) from a cohort of 57 individuals with obesity. The clinical characteristics of this cohort is summarized in **Table 1**. Macrophages and monocytes were identified based on their positive expression of CD45 and HLA-DR and lack of CD3, CD19, and CD56 expression (**Figure 1A**). Further, the macrophage population was divided into M1- (CD11c+CD206+) and M2-like (CD11c-CD206+) macrophages whereas monocytes were defined as CD11c+CD206- cells (**Figure 1B**). The total macrophage/monocyte population was larger in SAT compared to VAT (**Figure 1C**). However, the pro-inflammatory M1-like macrophage population was higher in VAT, whereas no differences were found in the amount ofM2-like macrophages between the two adipose tissue depots (**Figure 1D**). As expected, peripheral blood contained almost exclusively CD11c-positive monocytes, with a small, yet a detectable populations of CD11c+CD206+ myeloid cells (**Figure 1D**). Taken together, these data suggests that although SAT has the largest population of total monocytes and macrophages, pro-inflammatory M1-like macrophages are more abundant in VAT in individuals with obesity.

### Confirming the Pro- and Anti-Inflammatory Characteristics of M1-Like and M2-Like ATMs

Currently, there is a lack of consensus regarding which surface receptors best define M1- and M2-like macrophages in adipose tissue. We therefore investigated whether the stratification of macrophages into pro- and anti-inflammatory subsets based on expression of CD11c and CD206 could be further refined by assessing expression of other, known macrophage/monocyte surface receptors, such as CCR2, CD16, CD163, CD44, CD14, CD40, and HLA-DR (**Figure 1E**). Indeed, the M1-like macrophages (CD11c+ CD206+) expressed high levels of CCR2, an established pro-inflammatory, chemotactic marker (23), whereas CCR2-expression on the M2-like macrophages (CD11c-CD206+) was low (**Figure 1F**). CCR2 expression was also high on monocytes both in blood and adipose tissue. CD44 is another pro-inflammatory receptor (12) that displayed a similar pattern to CCR2, although with somewhat higher expression on M2-like macrophages compared to CCR2 (**Figure 1G**). CD14 and CD16 have also been described as pro-inflammatory ATM surface receptors (24). In line with this, we found CD16 expression on more of the M1-like macrophages than the M2-like macrophages. However, about 80% of the M1-like macrophages did not express CD16 (**Figure 1H**), suggesting the existence of CD16+/- M1-like macrophage subpopulations in both SAT and VAT. CD14 was more highly expressed on macrophages than on monocytes, but there was no difference in CD14 expression between the M1- and M2-like macrophage populations (**Figure 1I**). In agreement with their high capacity for antigen presentation, M1-like macrophages expressed the highest levels of HLA-DR in both fat depots and in the blood (**Figure 1J**). The same pattern was observed for CD40 (**Figure 1K**), which is expressed at higher levels on activated macrophages and is known to be expressed on recruited ATMs (24). CD163 has been described as an anti-inflammatory macrophage marker (25). Accordingly, CD163 was expressed on a higher percentage of the M2-like macrophages relative to the M1-like macrophages in VAT. However, this was not the case in SAT, and it should be noted that expression of CD163 varied considerably within both the M1- and M2-like cell populations (**Figure 1L**).

Taken together, the positive expression of CD44, HLA-DR, CD40, and in particular CCR2, support the pro-inflammatory identity of M1-like macrophages. M2-like macrophages displayed low levels of CCR2 and at the same time expressed somewhat higher levels of CD163 than M1-like macrophages, supporting their anti-inflammatory nature. The large variation in the expression of several surface receptors on both the M1- and the M2-like macrophages suggest that subpopulations within both pro- and anti-inflammatory ATMs exist, potentially with different functional patterns.

### Identification of Novel Adipose Tissue Macrophages Surface Proteins Using a Proteome Screen

**Figure 2:**
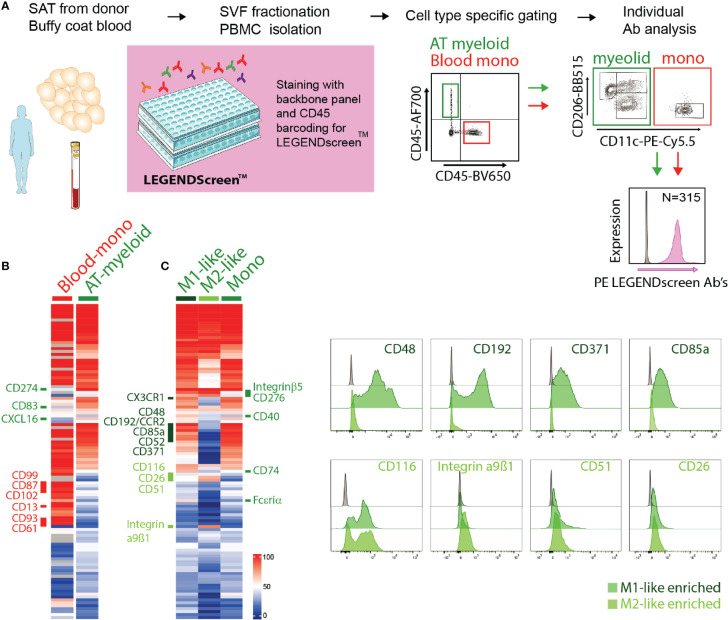
Surface proteome analysis of adipose tissue macrophages. **(A)** Experimental overview of the surface proteome screening using the LEGENDscreen™ kit. **(B)** Expression of surface proteins on blood monocytes (Blood-mono) and AT myeloid cells. Heatmap based on the percentage of cells expressing each surface protein, with a cut-off of minimum 20% expression by at least one of the populations. **(C)** Expression of surface proteins on AT M1- and M2-like macrophages and monocytes (mono). Heatmap based on the percentage of cells expressing each surface protein, with a cut-off of minimum 20% expression by at least one of the populations. Representative staining of a selection of surface proteins expressed on M1-like (dark green) or M2-like (light green) ATMs.

With the aim to identify novel surface receptor expression for detailed characterization of macrophages and monocytes in the adipose tissue, we performed a flow cytometry-based surface proteome screening using the LEGENDScreen™ kit. SVF isolated from SAT and PBMCs isolated from blood donors were pre-stained with a backbone panel allowing for the identification of myeloid cells from the AT as well as blood monocytes (CD45^+^CD14^+^HLA-DR^+^ cells), and then evaluated for surface expression of 315 distinct proteins. The experimental overview is presented in **Figure 2A**.

Among the commonly expressed surface markers by AT- and blood monocytes, the blood monocytes generally expressed higher levels of many of the analyzed markers (**Figure 2B**). Also, a number of surface proteins seemed to be unique for blood monocytes, including CD99, CD87, CD102, CD13, CD93 and CD61, whereas only three markers, CD274, CD83, and CXCL16, were unique to the AT monocytes (**Figure 2B**). It is worth noting that the blood and AT samples were not obtained from the same donors.

We further compared the expression of the surface proteins on AT monocytes (CD11c^+^CD206^-^), M1- (CD11c^+^CD206^+^), and M2-like (CD11c^-^CD206^+^) macrophages (**Figure 2C**). A substantial overlap was observed between the surface proteome of macrophages and monocytes and when comparing M1- and M2-like macrophages, but we also identified distinct M1- and M2-like macrophage and monocyte/macrophage proteins (**Figure 2C**). M1-like macrophages expressed the surface proteins CCR2, CD48, CD371, CD85a, CD49d, CX3CR1, and CD52 at higher levels than the M2-like macrophages, while CD116, CD51, CD26, and integrin α9β1 were expressed at higher levels on the M2-like macrophages. AT monocytes displayed a surface receptor expression pattern similar to the M1-like macrophages, still, several surface proteins were expressed on M1-like macrophages but not on AT monocytes, such as CD40, CD74, FcϵRIα, integrin β5, and CD276. The last two were also expressed on M2-like macrophages, indicating that these receptors separate ATMs from AT monocytes.

### Verification of Identified ATM Surface Proteins and Comparing Their Expression in SAT and in VAT

**Figure 3:**
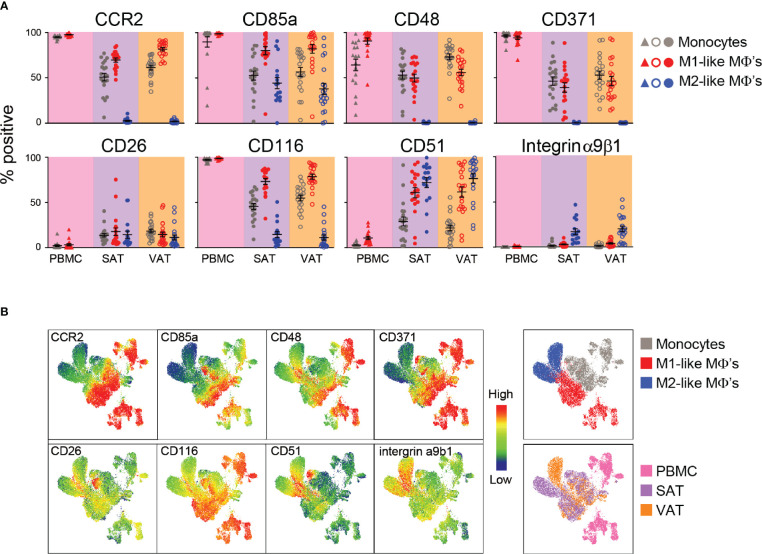
Verification of surface protein expression on M1- and M2-like adipose tissue macrophages from humans with obesity. **(A)** Scatter plots showing expression of surface proteins CCR2, CD85a, CD48, CD371, CD26, CD116, CD51, and integrin α9β1 on monocytes (grey), M1 (red)-, and M2 (blue)-like macrophages (MΦ’s) from PBMC (n=17), SAT (N=23), and VAT (n=23). **(B)** UMAP plots showing expression of the surface proteins analyzed in **(A)** with blue indicating low and red indicating high expression as well as UMAP plots colored according to cell origin (top) and tissue origin (bottom).

Based on degree of differential expression and novelty, we next selected some surface proteins for a more detailed characterization of macrophages from both subcutaneous and visceral adipose tissue in a new cohort of individuals with obesity (N=23). This allowed for validation of the protein expression on ATMs obtained from the proteomic screen as well as exploring the expression of these surface proteins on ATMs from individuals with obesity. We analyzed expression of the surface proteins CCR2/CD192, CD85a, CD48, and CD371, which were found to be highly expressed on M1-like macrophages and the proteins CD26, CD116, CD51 and integrin α9β1 that were expressed at higher levels on the M2-like macrophages. We confirmed that expression of CCR2 was high on M1-like macrophages and AT monocytes and low on M2-like macrophages (**Figure 3A**) and this protein was expressed at higher levels on M1-like macrophages from VAT compared to SAT. CD85a was also highly expressed on M1-like compared to M2-like macrophages, however, this protein was also expressed on a smaller population of M2-like macrophages (**Figure 3A**). CD48 and CD371, known to be involved in regulation and initiation of immune responses (26), were also expressed more highly on M1-like compared to M2-like macrophages (**Figure 3A**). However, on average only around 50% of M1-like macrophages expressed these proteins, and with expression varying from low (around 10%) to higher (around 80%), which could be indicative of subtypes of M1-like macrophages.

CD26, also known as dipeptidyl peptidase-4 (DPP-4), was expressed on around 70% of M2-like macrophages and 50% of M1-like macrophages from the screening. However, the validation showed that this protein was expressed at low levels on both M1- and M2-like macrophages, and expression was not different between the two ATM populations (**Figure 3A**). CD116, also identified as a M2-like specific marker from the screening, was expressed at higher levels on M1-like macrophages and was quite lowly expressed on M2-like macrophages, suggesting that this might be an M1-like rather than a M2-like macrophage marker (**Figure 3A**). Another surface protein found to be more highly expressed on M2-like macrophages in the screening was CD51 and this protein was expressed on higher levels on M2-like macrophages compared to M1-like macrophages, but only in VAT (**Figure 3A**). Integrin α9β1 was found to be highly expressed on M2-like macrophages, and a higher expression on M2-like compared to M1-like macrophages was confirmed in both SAT and VAT in the validation cohort (**Figure 3A**). However, the overall expression was found to be low on both M1- and M2-like macrophages in contrast to what was observed in the screening.

The single-parameter flow cytometry shown in **Figure 3A** gives an overall representation of each surface marker expression on the different cell types; however, it does not provide information about the combination of different surface proteins on each cell. Thus, we performed UMAP analysis to investigate multivariate relationships between the phenotypic markers (**Figure 3B**). To this end, monocytes, M1-, and M2-like macrophages from peripheral blood, SAT and VAT were electronically barcoded, concatenated, and analyzed. We observed that myeloid cells from peripheral blood (pink) clustered separately from the adipose tissue myeloid cells (purple and orange), indicating tissue-specific expression patterns. Also, separate clusters were observed for monocytes (grey) and M1-like macrophages (red) from the peripheral blood, which was also true for monocytes, M1- and M2-like macrophages in the adipose tissue. Here, the monocyte and M1-like macrophage clusters were closer together compared to the M2-like macrophages, indicating their similarities. Interestingly, the M2-like macrophages from SAT and VAT seemed to localize in two clusters, indicating that these cells might be phenotypically different depending on the adipose depot where they reside.

The high-dimensional UMAP analysis confirmed that CCR2 and CD116 were highly expressed on monocytes and M1-like macrophages and lowly expressed on M2-like macrophages. The expression patterns of CD48 and CD371 were similar, but highly expressed only in a subgroup of the M1-macrophages. The VAT M2-like macrophages displayed a higher expression of both integrin α9β1 and CD51, and there was a separate population of SAT M2-like macrophages that expressed higher levels of CD26, CD116, and CD51. The UMAP analysis also confirmed overall low expression of CD26. However, this protein seemed to be differentially expressed between distinct myeloid populations, including a group of monocytes that expressed high levels of CD116, CD26, and CD85a and low levels of CD48, further supporting the existence of monocyte/macrophage subsets.

Taken together, we verified CCR2, CD85a, CD48 and CD371 as proteins expressed at M1-like macrophages. Additionally, CD116 was found to be highly expressed on M1- compared to M2-like macrophages. Expression of CD26 was overall low but enriched in some clusters, whereas CD51 and integrin α9β1 seems to be distinctly expressed on M2-like macrophages.

### Adipose Tissue Macrophages Associate With Insulin Resistance and Circulating Lipids

**Figure 4:**
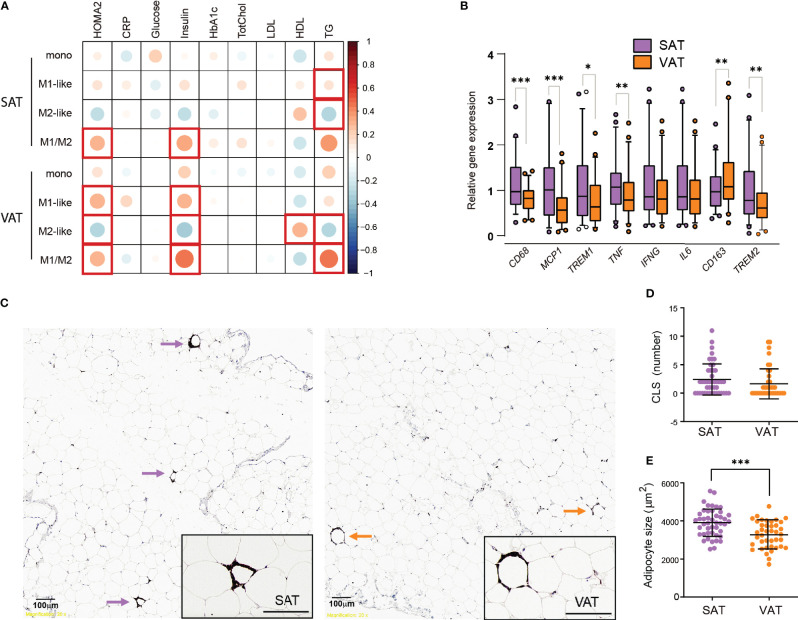
Insulin resistance, dyslipidemia, and inflammatory markers in SAT and VAT. **(A)** Pearson correlations between monocytes, M1-, and M2-like macrophages and M1/M2 ratio in SAT and VAT and biochemical parameters, corrected for BMI (n=80). Size and color intensities of circles indicate correlation coefficients. Red squares indicate significant (p<0.05) correlations. **(B)** Relative gene expression of *CD68*, *MCP1*, *TREM1*, *TNF*, *IFNG*, *IL6*, *CD163*, and *TREM2* for n = 46 (39 matched) SAT and VAT samples. Results are presented as fold changes (2^-ΔΔCT^) of target gene and normalized to *IPO8*. Expression levels of the SAT samples were set to 100%. **(C)** Representative pictures showing from SAT and VAT samples stained with CD68 antibody identifying crown-like structures (CLS) defined as three or more macrophages surrounding an adipocyte. **(D)** Graph showing numbers of CLS in SAT and VAT for n = 46 (39 matched) SAT and VAT samples. **(E)** Graph showing median adipocyte size in µm^2^ in SAT and VAT for n = 53 SAT and VAT samples. Bars in **(B)** represent mean and error bars represent SD. For **(D, E)** line represents the mean and error bars represents SD. The Wilcoxon matched pairs signed rank test was used for comparison between SAT and VAT in **(B, D)**. The paired t-test was used for comparison between SAT and VAT in **(E)**. *p < 0.05, **p < 0.01, ***p < 0.001.

Next, we wanted to investigate how the M1- and M2-like macrophage composition in the adipose tissue of individuals with obesity was related to systemic insulin resistance and other parameters relevant for metabolic function in the total patient cohort (Cohort 1 and 2, N=80). Insulin resistance, assessed by homeostatic model assessment for insulin resistance (HOMA-IR), was positively correlated with M1-like macrophages (*r=0.319, p=0.014*) and negatively correlated with M2-like macrophages (*r= –0.325, p=0.012*) in VAT, but not in SAT. Additionally, HOMA-IR was correlated with the pro-inflammatory M1/M2 ratio in VAT (*r=0.331, p=0.011*). Because BMI is a potential confounder for associations with metabolic parameters, we performed a partial correlation analysis (**Figure 4A**). The correlation between HOMA-IR and M1-like macrophages, M2-like macrophages and the M1/M2 ratio in VAT was still statistically significant after correcting for BMI. Additionally, the M1/M2 ratio in SAT now displayed a positive correlation with HOMA-IR. Further, serum triglyceride levels correlated negatively with M2-like macrophages and positively with the M1/M2 ratio in both adipose tissue depots, whereas HDL levels were positively correlated with M2-like macrophages in VAT. Interestingly, there were no significant correlations between ATMs and circulating CRP levels.

Taken together, these results indicate that, independently of BMI, insulin resistance is associated with an elevated ratio of M1- to M2-like macrophages in both SAT and VAT. M1/M2 ratio in both adipose tissue depots correlated positively with triglycerides, and this seemed to be driven by a negative correlation between M2-like macrophages and triglycerides.

### Insulin Resistance, CLS and Transcriptional Inflammatory Markers in SAT and VAT

In the first cohort of 57 individuals with obesity we next compared adipose tissue inflammation in SAT and VAT of individuals with obesity by measuring inflammatory gene expression and performing immunohistochemistry analyses of crown-like structures (CLS). The gene expression level of the general macrophage marker *CD68* was higher in SAT than in VAT (**Figure 4B**), in agreement with the flow cytometry data of elevated macrophage/monocyte levels in SAT (**Figure 1C**). The same was the case for *CCL-2*, encoding the cytokine MCP-1, which is associated with infiltration of monocytes (23). The pro-inflammatory genes *TNF* and *TREM1* were also expressed at higher levels in SAT compared to VAT. However, no differences between SAT and VAT were found in the expression of two other pro-inflammatory genes, *IL6* and *interferon* (*IFN*)*-γ*. The two anti-inflammatory genes analyzed showed opposite expression patterns to each other, with *TREM2* being elevated in SAT, whereas *CD163* expression was higher in VAT. Thus, these gene expression data did not reveal clear differences in inflammation between the AT depots.

Furthermore, we found that CLS, made up of CD68-positive macrophages surrounding adipocytes, were present in both SAT and in VAT (**Figure 4C**), similar to what has been observed by others (27). However, there were in general very few CLS observed in our cohort, and we found no significant differences in the amount of CLS between SAT and VAT (**Figure 4D**). CLS are expected to form around large, dying adipocytes, and we therefore performed histological measurements of adipocyte size. We found a higher median adipocyte size in SAT compared to VAT (**Figure 4E**), however, when investigating the association between gene expression, numbers of CLS, adipocyte size, and insulin resistance or circulating lipid parameters, we found only weak, non-significant correlations (data not shown).

Taken together, these data support larger adipocytes and somewhat higher expression of macrophage-related genes in SAT as compared to VAT. However, the lack of depot-specific differences in the number of CLS and the measured transcriptional inflammatory markers suggest that any difference in inflammatory tone between SAT and VAT in our cohort of individuals with obesity is moderate. Moreover, we found no clear associations between these markers of adipose tissue inflammation and circulating biochemical markers for metabolic dysregulation.

## Discussion

Recent investigations, including single-cell transcriptional studies, have revealed that adipose tissue harbor various macrophage subpopulations, including metabolically activated macrophages, and antioxidant macrophages (Mox) (19, 28, 29). Similar to the M1- and M2-like ATMs, these cells respond to changes in adipose tissue microenvironment during obesity, also displaying pro- and anti-inflammatory properties (19). However, the literature regarding which combination of surface proteins that define the different ATM subtypes in humans is still ambiguous. Here, we used a surface proteomics approach to identify novel proteins for a more detailed characterization of human M1- and M2-like ATMs, defined as myeloid cells that co-express CD11c and CD206 or CD206 only, respectively (11).

The pro-inflammatory nature of M1-like ATMs was first confirmed by their elevated expression of CCR2, CD44, HLA-DR and CD40 relative to M2-like cells. The proteomics screen and subsequent verification analysis, further identified CD85a, CD48, CD371, CX3CR1 and CD116 as additional M1-like macrophages-specific markers. To our knowledge, CD85a, CD48, and CD371 have not previously been reported to be expressed on ATMs. CD48 and CD371 are involved in regulation and initiation of immune responses (26, 30), and CD85a and CX3CR1 are known to be expressed on various myeloid cell types (31, 32). Indeed, all these markers were also expressed on monocytes, which is in agreement with known functional similarities between AT monocytes and M1-like macrophages, such as their ability to infiltrate into the AT (24, 31). Interestingly, the surface proteome screening demonstrated generally low expression of proteins on the tissue-resident M2 macrophages, and only the expression levels of the two integrins CD51 and integrin α9β1 were confirmed to be expressed at higher levels on the M2-compared to M1-like macrophages. Nevertheless, an UMAP analysis, which includes signals from all the surface markers in the panel simultaneously, revealed two distinct M2-like populations in SAT and VAT, respectively, possibly with functional differences. This emphasizes the potential of using multiple markers to characterize ATM composition with high resolution.

The literature concerning depot-specific differences in adipose tissue macrophage subtypes is contradictory. While some studies claim that infiltrating pro-inflammatory macrophages are most abundant in VAT (14, 17), others report higher levels of M1-like macrophages in SAT (11). In our study, the flow cytometry data indicated a higher relative abundance of total macrophages and monocytes in SAT, but still the pro-inflammatory M1 macrophages were more abundant in VAT, supporting previous findings that AT inflammation in splanchnic areas is of clinical relevance in obesity (15).

The pro-inflammatory M1/M2 ratio in both SAT and VAT correlated positively with HOMA-IR and circulating markers of dyslipidemia in our study, in agreement with the notion that inflammation in both these adipose tissue depots may contribute to metabolic dysfunction (33). These correlations were for most parts intact when correcting for BMI, suggesting that BMI variation among individuals with obesity is of minor importance for these associations. Interestingly, the significant correlations between adipose tissue M1/M2 ratio and circulating triglyceride levels seemed to be mainly driven by the inverse association with M2-like macrophages levels, suggesting a protective role for M2 macrophages in the development of dyslipidemia. This is in line with recent findings of accumulating anti-inflammatory, lipid-metabolizing macrophages in the adipose tissue, acting to prevent metabolic derangements during overnutrition (29). In fact, these lipid-associated macrophages (LAMs) formed CLS for lipid transfer into the macrophages in order to prevent adipocyte hypertrophy and loss of systemic lipid homeostasis under obesity conditions, a process that was dependent on Trem2 expression (29). Accordingly, others have shown that CLS contain anti-inflammatory Mox macrophages, characterized by expression of HO1 and Txnrd1 (31). In this sense, CLS occurrence in adipose tissue is not necessarily a measure of inflammation, and the low levels of CLS macrophages found in our study may thus represent a weakened protective mechanisms rather than low inflammation, although a more detailed characterized of these CLS will be necessary before making firm conclusions about their role in obesity-related adipose tissue inflammation.

Interestingly, a very recent study found that pro-inflammatory ATMs were not related to adipose tissue insulin resistance in humans (34). In that study, adipose tissue insulin resistance was elegantly measured, using state-of the-art tracer methods to determine the insulin concentration necessary to suppresses adipose tissue lipolysis by 50% (IC_50_). It was found that adipocyte size is the major driver for adipose tissue insulin resistance, and that any positive associations between adipose tissue inflammation and adipose tissue insulin resistance could be explained by the confounding effect of adipocyte hypertrophy (34). This was the case both when measuring inflammatory gene expression and pro- and anti-inflammatory ATMs by immunohistochemistry, defined as CD14- and CD206-positive cells, respectively. In fact, our transcriptional data supported this notion, as we did not find any significant association between adipose tissue inflammatory gene expression levels and HOMA-IR. However, the inflammatory gene expression levels represent an average from many cell types, and is less accurate than flow cytometry data that provides a distinct, high-resolution measure of macrophages *per se*. The flow cytometry data should thus be considered a more representative measurement for ATM status than both gene expression data and low-resolution IHC single-markers measurements (34). Of note, in our study the positive association between pro-inflammatory ATMs and HOMA-IR remained statistically significant even after correcting for adipocyte size, and our data is thus in line with the established idea that inflammatory mediators in the obese adipose tissue represent a mechanistic link that can lead to systemic insulin resistance (35). The discrepancy between the studies may, in addition to the abovementioned methodological issues, be explained by the different measures of insulin resistance; HOMA-IR reflects the balance between hepatic glucose output and insulin secretion by the β-cell (36), whereas the IC_50_ reflects lipolytic activity in the adipose tissue. Thus, further investigation of the role of ATMs in adipose tissue insulin resistance should be investigated using more high-resolution methods in future studies.

Therapeutic use of anti-inflammatory agents has been suggested for the treatment of metabolic disease (35). Indeed, targeting classical inflammatory molecules including IL-1, IL-6 and TNF have been shown to reduce the risk of diabetes and improve insulin sensitivity in some studies, although with variable effects (37–39). These treatments normally target circulating cytokines, even though interactions with paracrine/autocrine signaling within the tissue may be more relevant (40). Thus, targeting the local inflammation in the adipose tissue may be a valid treatment strategy, and ATMs are considered important targets for the treatment of chronic inflammation and obesity-related metabolic diseases (41, 42). It is, however, still not clear whether accumulation of pro-inflammatory ATMs is a cause or a consequence of insulin resistance, and to what extent immune cell infiltration and activation have beneficial or detrimental effects on adipose tissue homeostasis (29, 43, 44). Thus, interventional strategies focusing on ATMs will require a thorough understanding of the balance between beneficial versus pathological immune cell subsets and how they contribute to metabolic homeostasis. In conclusion, our study confirms a positive association between pro-inflammatory ATM ratio in both SAT and VAT and insulin resistance, measured by HOMA-IR. We also provide novel ATM surface markers that may enable detailed characterization and functional measurements, as well as act as potential targets for therapeutic manipulation of adipose tissue macrophages in the treatment of metabolic disease.

## Data Availability Statement

The original contributions presented in the study are included in the article/**Supplementary Material**. Further inquiries can be directed to the corresponding author.

## Ethics Statement

The studies involving human participants were reviewed and approved by Regional Etisk komité for forskningsetikk, Vest Norge (REK Vest). The patients/participants provided their written informed consent to participate in this study.

## Author Contributions

KS planned and performed experiments, acquired and analyzed data and wrote the manuscript. NS designed the flow cytometry panels, planned experiments and reviewed/edited the manuscript. MH planned and performed experiments and reviewed/edited the manuscript. ZK and VL performed experiments, acquired and analyzed data. LL-A analyzed data. MC contributed to the discussion, supervised the work and reviewed/edited the manuscript. IH acquired data and reviewed/edited the manuscript. CB and HN sampled the adipose tissue biopsies. PN provided reagents and funding and reviewed/edited the manuscript. GM and NB provided funding, contributed to the discussion, supervised the work and reviewed/edited the manuscript. JF designed the study, oversaw its conduction, researched data, provided funding, contributed to the discussion, and wrote the manuscript. All authors contributed to the article and approved the submitted version.

## Conflict of Interest

The authors declare that the research was conducted in the absence of any commercial or financial relationships that could be construed as a potential conflict of interest.

## Publisher’s Note

All claims expressed in this article are solely those of the authors and do not necessarily represent those of their affiliated organizations, or those of the publisher, the editors and the reviewers. Any product that may be evaluated in this article, or claim that may be made by its manufacturer, is not guaranteed or endorsed by the publisher.

## References

[1] Haslam DW James WPT. Obesity. Lancet (2005) 366(9492):1197–209. doi: 10.1016/S0140-6736(05)67483-1 16198769

[2] Hotamisligil GS. Inflammation and Metabolic Disorders. Nature (2006) 444(7121):860–7. doi: 10.1038/Nature05485 17167474

[3] Lumeng CN Saltiel AR. Inflammatory Links Between Obesity and Metabolic Disease. J Clin Invest (2011) 121(6):2111–7. doi: 10.1172/JCI57132 PMC3104776 21633179

[4] Kahn SE Hull RL Utzschneider KM. Mechanisms Linking Obesity to Insulin Resistance and Type 2 Diabetes. Nature (2006) 444(7121):840–6. doi: 10.1038/Nature05482 17167471

[5] Xu H Barnes GT Yang Q Tan G Yang D Chou CJ. Chronic Inflammation in Fat Plays a Crucial Role in the Development of Obesity-Related Insulin Resistance. J Clin Invest (2003) 112(12):1821–30. doi: 10.1172/JCI19451 PMC296998 14679177

[6] Weisberg SP McCann D Desai M Rosenbaum M Leibel RL Ferrante AW. Obesity is Associated With Macrophage Accumulation in Adipose Tissue. J Clin Invest (2003) 112(12):1796–808. doi: 10.1172/JCI19246 PMC296995 14679176

[7] Lumeng CN Bodzin JL Saltiel AR. Obesity Induces a Phenotypic Switch in Adipose Tissue Macrophage Polarization. J Clin Invest (2007) 117(1):175–84. doi: 10.1172/JCI29881 PMC1716210 17200717

[8] Fernø J Strand K Mellgren G Stiglund N Björkström NK. Natural Killer Cells as Sensors of Adipose Tissue Stress. Trends Endocrinol Metab (2020) 31(1):3–12. doi: 10.1016/j.Tem.2019.08.011 31597606

[9] Chávez-Galán L Olleros ML Vesin D Garcia I. Much More Than M1 and M2 Macrophages, There are Also CD169+ and TCR+ Macrophages. Front Immunol (2015) 6:263(MAY). doi: 10.3389/Fimmu.2015.00263 26074923 PMC4443739

[10] Bertani FR Mozetic P Fioramonti M Iuliani M Ribelli G Pantano F. Classification of M1/M2-Polarized Human Macrophages by Label-Free Hyperspectral Reflectance Confocal Microscopy and Multivariate Analysis. Sci Rep (2017) 7(1):8965. doi: 10.1038/S41598-017-08121-8 28827726 PMC5566322

[11] Wentworth JM Naselli G Brown WA Doyle L Phipson B Smyth GK. Pro-Inflammatory CD11c+CD206+ Adipose Tissue Macrophages Are Associated With Insulin Resistance in Human Obesity. Diabetes (2010) 59(7):1648–56. doi: 10.2337/Db09-0287 PMC2889764 20357360

[12] Liu LF Kodama K Wei K Tolentino LL Choi O Engleman EG. The Receptor CD44 is Associated With Systemic Insulin Resistance and Proinflammatory Macrophages in Human Adipose Tissue. Diabetologia (2015) 58(7):1579–86. doi: 10.1007/S00125-015-3603-Y 25952479

[13] Apovian CM Bigornia S Mott M Meyers MR Ulloor J Gagua M. Adipose Macrophage Infiltration is Associated With Insulin Resistance and Vascular Endothelial Dysfunction in Obese Subjects. Arterioscler Thromb Vasc Biol (2008) 28(9):1654–9. doi: 10.1161/ATVBAHA.108.170316 PMC2728436 18566296

[14] Harman-Boehm I Bl̈her M Redel H Sion-Vardy N Ovadia S Avinoach E. Macrophage Infiltration Into Omental Versus Subcutaneous Fat Across Different Populations: Effect of Regional Adiposity and the Comorbidities of Obesity. J Clin Endocrinol Metab (2007) 92(6):2240–7. doi: 10.1210/Jc.2006-1811 17374712

[15] Klöting N Fasshauer M Dietrich A Kovacs P Schön MR Kern M. Insulin-Sensitive Obesity. Am J Physiol Metab (2010) 299(3):E506–15. doi: 10.1152/Ajpendo.00586.2009 20570822

[16] Appleton SL Seaborn CJ Visvanathan R Hill CL Gill TK Taylor AW. Diabetes and Cardiovascular Disease Outcomes in the Metabolically Healthy Obese Phenotype. Diabetes Care (2013) 36(8):2388–94. doi: 10.2337/Dc12-1971 PMC3714523 23491523

[17] Cancello R Tordjman J Poitou C Guilhem G Bouillot JL Hugol D. Increased Infiltration of Macrophages in Omental Adipose Tissue Is Associated With Marked Hepatic Lesions in Morbid Human Obesity. Diabetes (2006) 55(6):1554–61. doi: 10.2337/Db06-0133 16731817

[18] Hardy OT Perugini RA Nicoloro SM Gallagher-Dorval K Puri V Straubhaar J. Body Mass Index-Independent Inflammation in Omental Adipose Tissue Associated With Insulin Resistance in Morbid Obesity. Surg Obes Relat Dis (2011) 7(1):60–7. doi: 10.1016/j.Soard.2010.05.013 PMC2980798 20678967

[19] Kratz M Coats BR Hisert KB Hagman D Mutskov V Peris E. Metabolic Dysfunction Drives a Mechanistically Distinct Proinflammatory Phenotype in Adipose Tissue Macrophages. Cell Metab (2014) 20(4):614–25. doi: 10.1016/j.Cmet.2014.08.010 PMC4192131 25242226

[20] Hill DA Lim H-W Kim YH Ho WY Foong YH Nelson VL. Distinct Macrophage Populations Direct Inflammatory Versus Physiological Changes in Adipose Tissue. Proc Natl Acad Sci (2018) 115(22):E5096–105. doi: 10.1073/Pnas.1802611115 PMC5984532 29760084

[21] Levy JC Matthews DR Hermans MP. Correct Homeostasis Model Assessment (HOMA) Evaluation Uses the Computer Program. Diabetes Care (1998) 21(12):2191–2. doi: 10.2337/Diacare.21.12.2191 9839117

[22] Galarraga M Campión J Muñoz-Barrutia A Boqué N Moreno H Martínez JA. Adiposoft: Automated Software for the Analysis of White Adipose Tissue Cellularity in Histological Sections. J Lipid Res (2012) 53(12):2791–6. doi: 10.1194/Jlr.D023788 PMC3494244 22993232

[23] Weisberg SP Hunter D Huber R Lemieux J Slaymaker S Vaddi K. CCR2 Modulates Inflammatory and Metabolic Effects of High-Fat Feeding. J Clin Invest (2006) 116(1):115–24. doi: 10.1172/JCI24335 PMC1307559 16341265

[24] Russo L Lumeng CN. Properties and Functions of Adipose Tissue Macrophages in Obesity. Immunology (2018) 155(4):407–17. doi: 10.1111/Imm.13002 PMC6230999 30229891

[25] Zeyda M Farmer D Todoric J Aszmann O Speiser M Györi G. Human Adipose Tissue Macrophages Are of an Anti-Inflammatory Phenotype But Capable of Excessive Pro-Inflammatory Mediator Production. Int J Obes (2007) 31(9):1420–8. doi: 10.1038/Sj.Ijo.0803632 17593905

[26] Zou C Zhu C Guan G Guo Q Liu T Shen S. CD48 is a Key Molecule of Immunomodulation Affecting Prognosis in Glioma. Onco Targets Ther (2019) 12:4181–93. doi: 10.2147/OTT.S198762 PMC6549391 31213836

[27] Cinti S Mitchell G Barbatelli G Murano I Ceresi E Faloia E. Adipocyte Death Defines Macrophage Localization and Function in Adipose Tissue of Obese Mice and Humans. J Lipid Res (2005) 46(11):2347–55. doi: 10.1194/Jlr.M500294-JLR200 16150820

[28] Kadl A Meher AK Sharma PR Lee MY Doran AC Johnstone SR. Identification of a Novel Macrophage Phenotype That Develops in Response to Atherogenic Phospholipids *via* Nrf2. Circ Res (2010) 107(6):737–46. doi: 10.1161/CIRCRESAHA.109.215715 PMC2941538 20651288

[29] Jaitin DA Adlung L Thaiss CA Weiner A Li B Descamps H. Lipid-Associated Macrophages Control Metabolic Homeostasis in a Trem2-Dependent Manner. Cell (2019) 178(3):686–98.e14. doi: 10.1016/j.Cell.2019.05.054 31257031 PMC7068689

[30] Yan H Kamiya T Suabjakyong P Tsuji NM. Targeting C-Type Lectin Receptors for Cancer Immunity. Front Immunol (2015) 6:408. doi: 10.3389/Fimmu.2015.00408 26379663 PMC4547497

[31] Serbulea V Upchurch CM Schappe MS Voigt P DeWeese DE Desai BN. Macrophage Phenotype and Bioenergetics are Controlled by Oxidized Phospholipids Identified in Lean and Obese Adipose Tissue. Proc Natl Acad Sci (2018) 115(27):E6254–63. doi: 10.1073/Pnas.1800544115 PMC6142199 29891687

[32] Yeboah MJ Papagregoriou C Jones DC Chan HTC Hu G McPartlan JS. LILRB3 (ILT5) Is a Myeloid Cell Checkpoint That Elicits Profound Immunomodulation. JCI Insight (2020) 5(18):e141593. doi: 10.1172/Jci.Insight.141593 PMC7526549 32870822

[33] Burhans MS Hagman DK Kuzma JN Schmidt KA Kratz M. Contribution of Adipose Tissue Inflammation to the Development of Type 2 Diabetes Mellitus. Compr Physiol (2018) 9(1):1–58. doi: 10.1002/Cphy.C170040. Wiley; Wiley. 30549014 PMC6557583

[34] Espinosa De Ycaza AE Søndergaard E Morgan-Bathke M Lytle K Delivanis DA Ramos P. Adipose Tissue Inflammation Is Not Related to Adipose Insulin Resistance in Humans. Diabetes (2021) 71(3):381–93. doi: 10.2337/DB21-0609 PMC8893944 34857544

[35] Hotamisligil GS. Inflammation, Metaflammation and Immunometabolic Disorders. Nature (2017) 542(7640):177–85. doi: 10.1038/NATURE21363 28179656

[36] Wallace TM Levy JC Matthews DR. Use and Abuse of HOMA Modeling. Diabetes Care (2004) 27(6):1487–95. doi: 10.2337/Diacare.27.6.1487 15161807

[37] Larsen CM Faulenbach M Vaag A Vølund A Ehses JA Seifert B. Interleukin-1-Receptor Antagonist in Type 2 Diabetes Mellitus. N Engl J Med (2007) 356(15):1517–26. doi: 10.1056/NEJMOA065213 17429083

[38] Solomon DH Massarotti E Garg R Liu J Canning C Schneeweiss S. Association Between Disease-Modifying Antirheumatic Drugs and Diabetes Risk in Patients With Rheumatoid Arthritis and Psoriasis. JAMA (2011) 305(24):2525–31. doi: 10.1001/JAMA.2011.878 21693740

[39] Burska AN Sakthiswary R Sattar N. Effects of Tumour Necrosis Factor Antagonists on Insulin Sensitivity/Resistance in Rheumatoid Arthritis: A Systematic Review and Meta-Analysis. PloS One (2015) 10:6. doi: 10.1371/JOURNAL.PONE.0128889 PMC4482317 26110878

[40] Sethi JK Hotamisligil GS. Metabolic Messengers: Tumour Necrosis Factor. Nat Metab (2021) 3(10):1302–12. doi: 10.1038/S42255-021-00470-Z 34650277

[41] Ma L Liu TW Wallig MA Dobrucki IT Dobrucki LW Nelson ER. Efficient Targeting of Adipose Tissue Macrophages in Obesity With Polysaccharide Nanocarriers. ACS Nano (2016) 10(7):6952–62. doi: 10.1021/ACSNANO.6B02878 27281538

[42] Peterson KR Cottam MA Kennedy AJ Hasty AH. Macrophage-Targeted Therapeutics for Metabolic Disease. Trends Pharmacol Sci (2018) 39(6):536–46. doi: 10.1016/J.TIPS.2018.03.001 PMC5962426 29628274

[43] Czech MP. Insulin Action and Resistance in Obesity and Type 2 Diabetes. Nat Med (2017) 23(7):804–14. doi: 10.1038/NM.4350 PMC6048953 28697184

[44] Shimobayashi M Albert V Woelnerhanssen B Frei IC Weissenberger D Meyer-Gerspach AC. Insulin Resistance Causes Inflammation in Adipose Tissue. J Clin Invest (2018) 128(4):1538–50. doi: 10.1172/JCI96139 PMC5873875 29528335

