# Supplementary material for: Subtype-Specific Surface Proteins on Adipose Tissue Macrophages and Their Association to Obesity-Induced Insulin Resistance
Source: Front Endocrinol (Lausanne). 2022 Apr 11;13:856530. doi: 10.3389/fendo.2022.856530 (PMC9035670; doi:10.3389/fendo.2022.856530)
Supplement: Supplementary file 1 [file DataSheet_1.docx]

**Table S1 List of flow cytometry antibodies**

| Antibody | Manufacturer | Catalogue number |
| --- | --- | --- |
| CD3 PE-Cy5 | Biolegend | 300410 |
| CD11c PE-Cy5.5 | Life Technologies | MHCD11c18 |
| CD11c PE-Cy7 | Biolegend | 337215 |
| CD14 BV605 | Biolegend | 301834 |
| CD16 BV711 | BD Biosciences | 563127 |
| CD19 PE-Cy5 | Biolegend | 302210 |
| CD40 PE | Biolegend | 313006 |
| CD44 PE-CF594 | BD Biosciences | 562818 |
| CD45 AF700 | Biolegend | 304024 |
| CD48 PE-CF594 | BD Biosciences | 562717 |
| CD51 PE-Cy7 | Biolegend | 327915 |
| CD56 PE-Cy5 | Biolegend | 304607 |
| CD85a AF647 | BD Biosciences | 564469 |
| CD116 BV650 | BD Biosciences | 564044 |
| CD163 AF647 | BD Biosciences | 562669 |
| CD192 (CCR2) BV421 | Biolegend | 357209 |
| CD206 BB515 | BD Biosciences | 564668 |
| CD371 BB700 | BD Biosciences | 746224 |
| HLA-DR APC-Cyanine7 | Biolegend | 307617 |
| Integrin α9β1 PE | Biolegend | 351605 |

**Table S2 List of primers for qPCR**

| Gene  Primer name | Forward | Reverse |
| --- | --- | --- |
| *CD68* | CCACACAGGGGTCTTTGG | GATGAGAGGCAGCAAGATGG |
| *CD163* | GAAGATGCTGGCGTGACAT | GCTGCCTCCACCTCTAAGTC |
| *IL6* | ATAGGACTGGAGATGTCTGAGG | GCTTGTGGAGAAGGAGTTCATAG |
| *INFγ* | GAACTCTTTTCTTAGGCATTTTGAAG | CACTCTTTTGGATGCTCTGGT |
| *IPO8* | CGGATTATAGTCTCTGACCATGTG | TGTGTCACCATGTTCTTCAGG |
| *MCP1* | AGTCTCTGCCGCCCTTCT | GTGACTGGGGCATTGATTG |
| *TNF* | CAGCCTCTTCTCCTTCCTGAT | GCCAGAGGGCTGATTAGAGA |
| *TREM1* | AGTTACAGCCCAAAACATGC | CAGCCCCCACAAGAGAATTA |
| *TREM2* | ACAGAAGCCAGGGACACATC | CCTCCCATCATCTTCCTTCA |
